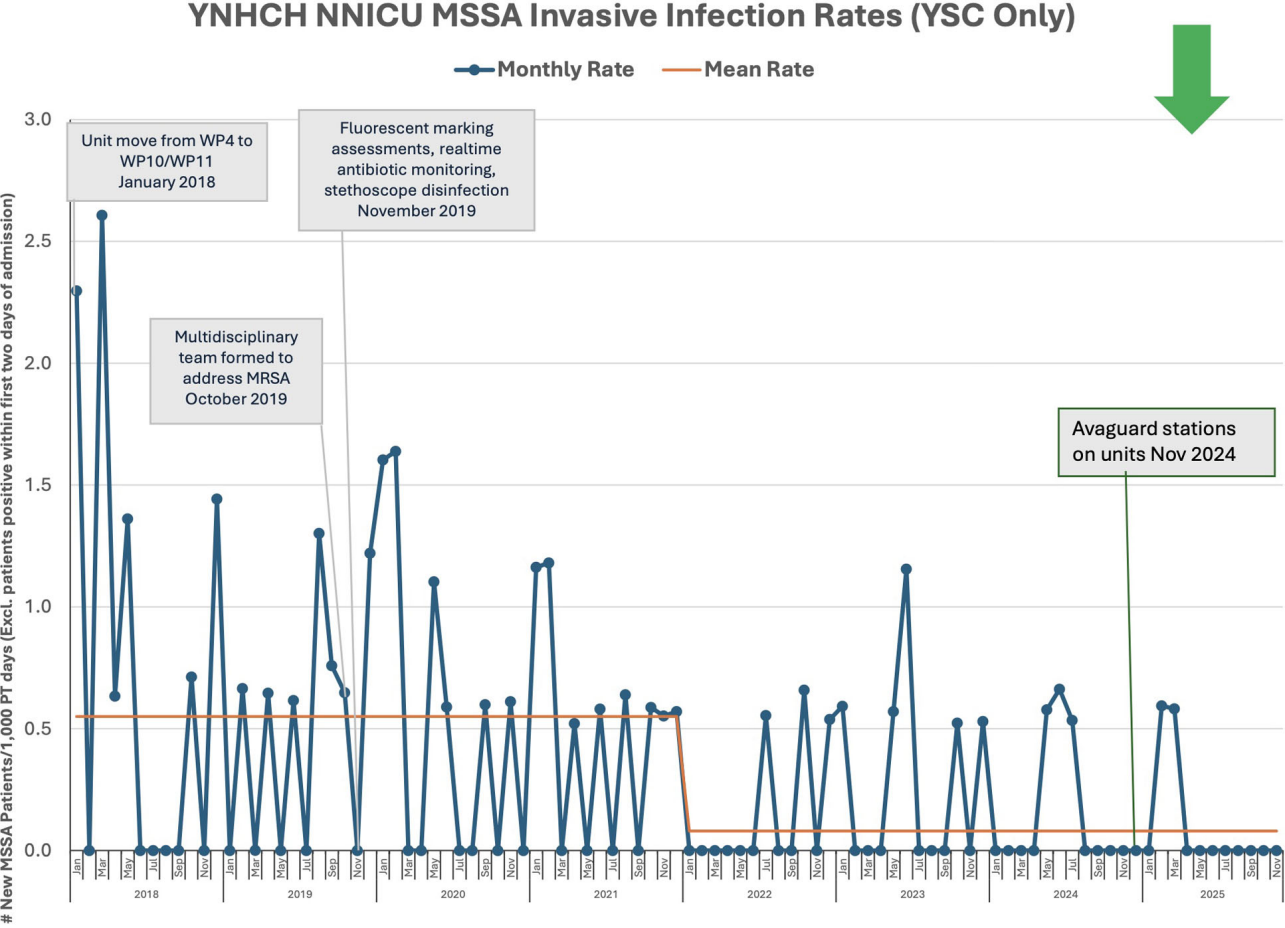# 345 Patient and Provider Differences between Treated and Untreated Cases of PCR (+)/Toxin EIA (-) clostridioides difficile Infections

**DOI:** 10.1017/ash.2026.10685

**Published:** 2026-06-23

**Authors:** Tom Murray, Jayson Wright, Matthew Bizzarro, Noa Fleiss, Kathy Krechevsky, David Peaper

**Affiliations:** 1 Yale New Haven Children’s Hospital; 2 Yale School of Medicine; 3 Yale University School of Medicine; 4 Yale University; 5 Yale New Haven Health System

## Abstract

**Background:** Invasive Staphylococcus aureus (SA) infections are a common cause of sepsis in premature infants admitted to the neonatal intensive care unit (NICU). While a quality improvement team effectively reduced methicillin resistant (MRSA) invasive infections, methicillin susceptible (MSSA) infections remained problematic. As a result, there was a renewed effort to understand MSSA infection transmission and reduce invasive MSSA infections. **Methods:** The primary outcome was invasive MSSA infection, most commonly bacteremia, in the NICU. Environmental cultures identified MSSA on high touch point areas and whole genome sequencing (WGS) confirmed there were both endemic strains and independently introduced strains. New interventions to reduce invasive SA infections included additional environmental cleaning of clinical workstations with effectiveness measured by fluorescent marking and a start of shift chlorhexidine (CHG) /alcohol (Avaguard) below the elbow wash for NICU staff. Babies were screened weekly for MSSA/MRSA colonization. **Results:** In 2023 and 2024 when there were no MRSA infections, there were nine MSSA infections (Figure 1). WGS confirmed that environmental MSSA strains were recovered from infants. Increased screening for MSSA with selective decolonization with mupirocin for high risk infants did not temporally change invasive infection rates and the mupA gene for high level mupirocin resistance was identified in a subset of colonizing strains. There were no significant changes in rates of new colonization for either MSSA or MRSA in 2025. After 1697 days there was one MRSA infection in September 2025. Introducing enhanced environmental cleaning and below the elbow disinfection for staff was temporally associated with a reduction in invasive MSSA infections with only two MSSA infections in 2025, most recently in March. As of January 1, 2026 the NICU has gone nine months without a MSSA infection. **Conclusions:** Increased environmental cleaning of clinical workstations with a start of shift below the elbow staff disinfection has resulted in a sustained reduction in invasive MSSA infection without a significant reduction in colonization rates. Additional work is ongoing to further understand if SA environmental colonization is reduced and whether current strains colonizing infants are identical to those that have caused invasive infection in the past.

**Figure:**